# Annual Report on the Progress of Medicine in General in All Countries

**Published:** 1843-07

**Authors:** 


Art. IV.—*Jahresbericht über die Fortschritte der gesammten Medicin in allen Ländern.* Herausgegeben von Dr. C. Canstatt. Erstes u. zweites Heft.—*Erlangen,* 1842. 8vo.

*Annual Report on the Progress of Medicine in general in all Countries.* Edited by Dr. C. Canstatt. Parts I. II.—*Erlangen,* 1842.

These portions of Dr. Canstatt's report are sufficient to show that both in design and in execution it merits the highest place among the numerous systematic *reports,* which it has of late become customary to publish. The whole work is divided into no less than thirty-six departments, each of which is assigned to a different contributor who has made that part of medical science an object of particular study. Among the names of the contributors are several well known and distinguished ; for instance, Simon, to whom is assigned the department of medical chemistry and toxicology ; Albers, who takes that of pathological anatomy ; Remak, who has that of physiology ; Lessing, who has the history of medicines ; Stilling, who reports the progress of all relating to the nerves, &c. The parts already published are completely and, on the whole, well done ; though sometimes in striving not to omit anything, the fault is committed of slurring too quickly over the more important matters. There is also this more serious fault, that before the report arrives in England, the observations upon which it is made are two or more years old. It is therefore of comparatively little value to those who are anxious to be *au courant* with the progress of medical science, without the labour of wading through the multitude of journals that are now published. As an historical record, however, and as a book of reference, the work is excellent.

